# Emergence of rabbit haemorrhagic disease virus 2 in China in 2020

**DOI:** 10.1002/vms3.332

**Published:** 2020-08-02

**Authors:** Bo Hu, Houjun Wei, Zhiyu Fan, Yanhua Song, Mengmeng Chen, Rulong Qiu, Weifeng Zhu, Weizhong Xu, Jiabin Xue, Fang Wang

**Affiliations:** ^1^ Jiangsu Academy of Agricultural Sciences Key Laboratory of Veterinary Biologicals Engineering and Technology Ministry of Agriculture National Center for Engineering Research of Veterinary Bio‐Products Institute of Veterinary Medicine Nanjing China

**Keywords:** China, *Lagovirus*, rabbit haemorrhagic disease virus, rabbits, RHDV2

## Abstract

Rabbit haemorrhagic disease (RHD) is an acute fatal disease caused by the *Lagovirus* rabbit haemorrhagic disease virus (RHDV), which was first reported in 1984 in China. Strains of two different genotypes (GI.1a and GI.1c) have been detected in China to date. In 2010, a new RHDV variant with a unique genetic and antigenic profile was identified in France, designated RHDV2, which rapidly spread throughout continental Europe and nearby islands. Here, we report the first outbreak of RHD induced by RHDV2 (GI.2) in rabbit farms in the Sichuan province of China. We conducted haemagglutination tests and phylogenetic analysis of the new RHDV isolate SC2020/04, which was identified as a non‐haemagglutinating strain belonging to the RHDV2 (GI.2) genogroup. Considering the serious risk of RHDV2 to the Chinese rabbit industry, the circulation of RHDV2 in the population should be carefully monitored in China.

## Graphical abstract

The first outbreak of rabbit hemorrhagic disease induced by rabbit hemorrhagic disease virus 2 (GI.2) in China.

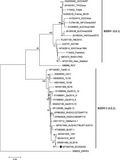

## 1 INTRODUCTION

China is highly ranked in the global rabbit industry, accounting for 43% of the worldwide slaughtered rabbits with 44% of the global share of rabbit meat output (Wu, Seema, & Huang, 2016). Rabbit haemorrhagic disease virus (RHDV) of the family *Caliciviridae*, genus *Lagovirus*, causes high morbidity and mortality in rabbits. Over 90% of RHDV‐infected adult rabbits die owing to fulminant hepatic failure within 3 days of infection (Park, Lee, & Itakura, 1995). RHDV was first reported in China in 1984. However, a new RHDV‐related virus designated RHDV2 was detected, for the first time, in France in 2010 (Le Gall‐Recule et al., 2013), and subsequently spread to other countries in Europe, Australia, America and Africa (Abrantes et al., 2013; Lopes, Rouco, Esteves, & Abrantes, 2019; Mahar et al., 2018; Puggioni et al., 2013; Rouco et al., 2018).

Based on phylogenetic analyses of RHDV VP60 sequences, RHDV was divided into classical RHDV G1‐G5 and G6 or RHDVa (Le Gall‐Recule et al., 2003) and the new strain named RHDV2 (Le Gall‐Recule et al., 2013). In 2017, a new RHDV nomenclature was proposed that changed G1, G2, G3‐G5 and G6 to GI.1b, GI.1c, GI.1d and GI.1a, respectively, and RHDV2 was called GI.2 (Le Pendu et al., 2017). To date, only two RHDV genotypes were known to be present in China, G2 (GI.1a) and G6 (GI.1c; Hu et al., 2017). Here, we report a new RHDV isolate collected from three infected rabbits at farms in the Sichuan province of China in April 2020, representing the first report of GI.2 in China.

## 2 MATERIALS AND METHODS

### 2.1 Haemagglutination test

Liver samples collected from three infected rabbits were frozen and stored at −70°C. Liver samples were homogenized (20% in phosphate‐buffered saline [PBS]), frozen at −70°C and thawed twice. The haemagglutination test (Hu et al., 2016) was carried out in U‐shaped microtitre plates containing 50 μl of PBS (pH 6.5). Fifty‐microlitre suspensions of homogenized liver samples were twofold serially diluted and placed in U‐shaped plates; they were then further incubated with an equal volume of 1% human O erythrocytes at 4°C, 25°C or 37°C. The haemagglutination was visually determined 30 min later. RHDV isolate WF/China/2007 (GenBank accession number: FJ794180) was used as a positive control.

### 2.2 Reverse transcription‐polymerase chain reaction

The full‐length of the *vp60* gene sequence was amplified by Reverse transcription‐polymerase chain reaction (RT‐PCR) using the Reverse Transcriptase XL (AMV) kit (Takara Bio) and the Ex Taq kit (Takara Bio). A specific primer pair (sense, 5'‐AAGAGAGTCGTCTCGGTAGTA‐3' and antisense 5'‐GCGCCTGCAAGTCCCAATCC‐3') was used as described previously (Duarte et al., 2014). The *vp60* gene was then cloned into a pMD‐19T vector (Takara Bio). Positive clones were sequenced and analysed further.

### 2.3 Phylogenetic analysis

Phylogenetic analysis of *vp60* gene sequences was performed using MEGA 7 (Kumar, Stecher, & Tamura, 2016) with the maximum‐likelihood approach based on the Kimura two‐parameter model. Reliability of the nodes was assessed with a bootstrap resampling procedure consisting of 1,000 replicates.

## 3 RESULTS AND DISCUSSION

The clinical symptoms and pathological changes in the dead rabbits were similar to those of rabbit haemorrhagic disease. The mortality rate was more than 70% (approximately 1,300 rabbits died), although weaning rabbits had been immunized with a commercial inactivated RHD vaccine. Importantly, most of the unweaned rabbits died of the disease, indicating that RHDV2 might be the causal pathogen because RHDV2 is able to fatally affect a high proportion of young rabbits.

Compared with the positive control, the new isolate failed to haemagglutinate at 4, 25 and 37 (haemagglutination titres ≤ 1:20). Given that the haemagglutination test remains the routine diagnostic method for RHDV in China, this non‐haemagglutinating characteristic warrants further attention in the detection of clinical samples.

**FIGURE 1:**
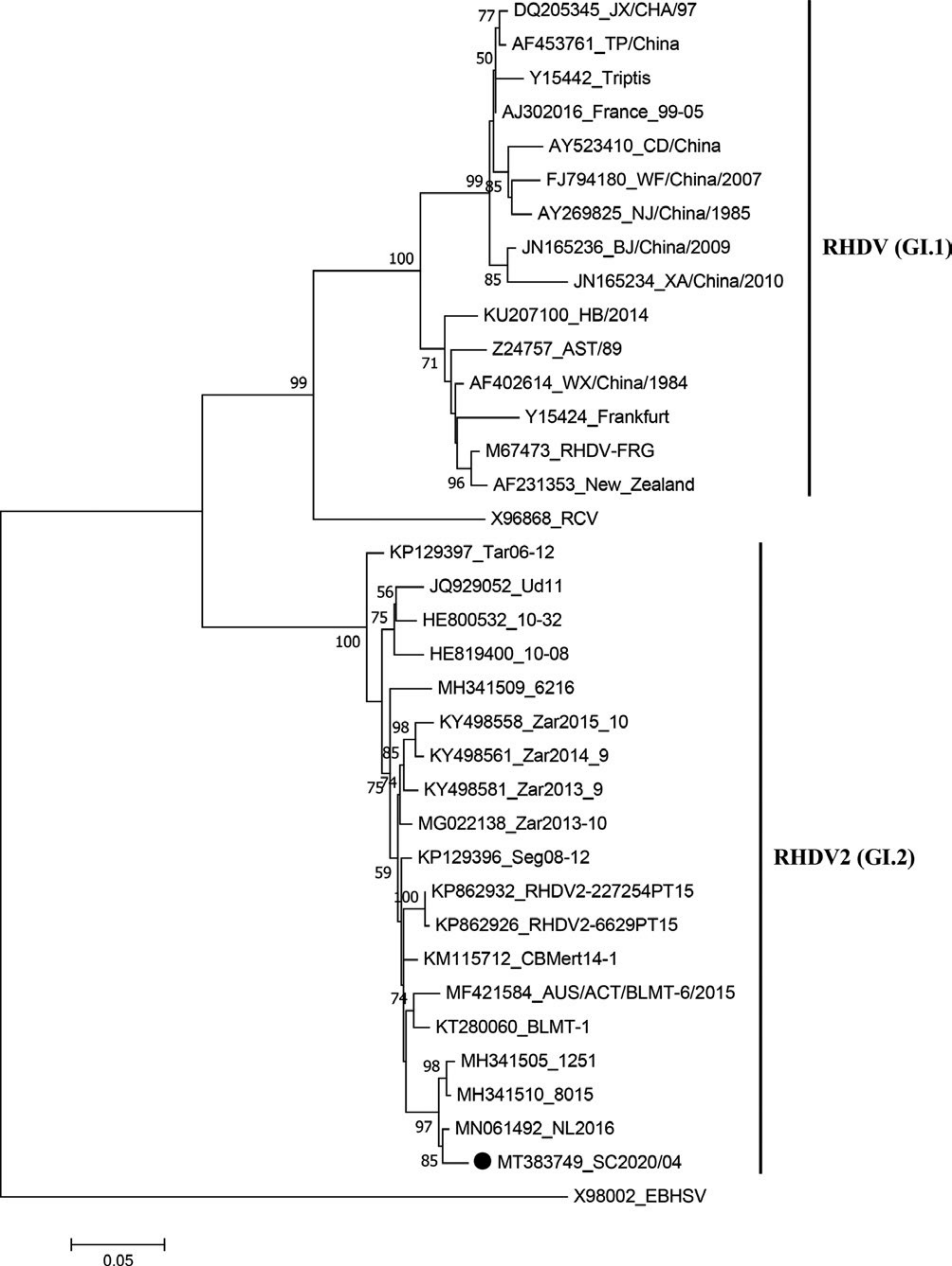
Maximum‐likelihood phylogenetic trees for the complete nucleotide sequences of RHDV *vp60* genes. Bootstrap probability values above 50% with 1,000 replicates are indicated at the nodes. The branch lengths are proportional to the genetic distance. European brown hare syndrome virus (EBHSV) strain BS89 was used as the outgroup to root the tree

The new isolate exhibits the highest nucleotide sequence identity with the NL2016 strain from the Netherlands (98.3%; GenBank accession number: MN061492), which corresponds to RHDV2. Phylogenetic analysis was employed to determine the evolution of the new isolate. As shown in Figure 1, the new isolate is in the same branch of the other RHDV2 strains. These results support the conclusion that the isolate collected from the Sichuan province of China in 2020 belongs to the RHDV2 (GI.2) genogroup, which was designated strain SC2020/04 (GenBank accession number: MT383749). This represents the first outbreak of RHDV2‐induced RHD in rabbit farms in China. We previously classified all RHDV isolates in China collected before 2017 in GI.1 (Hu et al., 2017); therefore, the present finding indicates the potential for co‐circulation of RHDV and RHDV2 in China. Indeed, RHDV2 (GI.2) was reported to replace RHDV (GI.1) in some countries, including Portugal, Sweden and Australia (Lopes et al., 2014; Mahar et al., 2018; Neimanis et al., 2018). In addition, recombinant events between GI.2 and other genotypes have been reported (Almeida et al., 2015; Lopes et al., 2015; Silverio et al., 2018). Considering the distinct serotype from RHDV (GI.1), high risk of RHDV2 (GI.2) to the Chinese rabbit industry, and limited level of cross protection induced by RHDV/RHDVa vaccine against RHDV2, ongoing surveillance and vaccine formulation update are most imminent requirements for control of the disease induced by RHDV2 in China.

## CONFLICT OF INTEREST

The authors declare no conflict of interest regarding the publication of this manuscript. All authors have read and approved the final manuscript.

## AUTHOR CONTRIBUTION

**Bo Hu:** Investigation; Writing‐original draft. **Houjun Wei:** Investigation; Writing‐review & editing. **Zhiyu Fan:** Investigation; Writing‐review & editing. **Yanhua Song:** Writing‐review & editing. **Mengmeng Chen:** Writing‐review & editing. **Rulong Qiu:** Software. **Weifeng Zhu:** Writing‐review & editing. **Weizhong Xu:** Investigation. **Jiabin Xue:** Investigation. **Fang Wang:** Conceptualization; Resources; Writing‐review & editing.

## ETHICAL APPROVAL

The authors confirm that the ethical policies of the journal have been adhered to. The collection of the liver samples was performed in strict accordance with the guidelines of Jiangsu Province Animal Regulations (Government Decree No. 45).

### PEER REVIEW

The peer review history for this article is available at https://publons.com/publon/10.1002/vms3.332.

## Data Availability

The data that support the findings of this study are available from the corresponding author upon reasonable request.
